# Teachers’ mental health literacy and action competencies: Julia Dratva

**DOI:** 10.1093/eurpub/ckac129.250

**Published:** 2022-10-25

**Authors:** J Dratva, M Kerry, K Albermann, D Robin

**Affiliations:** 1 Institute for Public Health, ZHAW Zürich University of Applied Sciences, Winterthur, Switzerland; 2 Social Pediatric Center SPZ, Winterthur Cantonal Hospital, Winterthur, Switzerland

## Abstract

**Background:**

Teachers are very important in mental health promotion and early recognition of mental health burden (disorder, illness). Teachers’ surrogate mental health literacy (MHL) may be key to improving mental health in youth but has been little investigated. We assessed surrogate MHL in Swiss teachers and tested a measure of mental health action competencies (MHAC).

**Methods:**

In 2020, all teaching and support staff at compulsory school level were invited to an online survey covering individual and professional characteristics, MHL (finding, understanding, critical appraisal of information) and action competencies (adapted scale Ahnert et al. 2016, range 17 - 68) personal experience with students’ MHB. Data was explored descriptively and with multivariate regression. Item response theory analyses were conducted to examine internal psychometric MHAC scale properties, and group-mean differences tested between school levels.

**Results:**

Participation rate was 38% (N = 459). Nearly all participants had taught at least 1 mentally burdened student in the past year (average 4.7). 77% felt experienced to very experienced regarding these students. Only 32% felt they had sufficient tools and teaching resources. Participants felt it was difficult to very difficult to find (47%), understand (53%) and appraise (90%) information on students’ mental health. Kindergarden teachers and teachers without class responsibility showed significantly lower MHL. Internal psychometric properties of the MHAC measure support the use of a 1-factor scale and indicates discriminant validity with respect to age; experience and school level, median score was high (P50 48, P25 44, P75 53), but single items, e.g. on suicide signs, were rated low.

**Conclusions:**

While overall subjective MHAC are high, teachers are insecure regarding MHL and report a lack of tools and resources. Targeted training could strengthen surrogate mental health literacy with a focus on critical appraisal and certain action competencies.

